# Extracellular vesicles derived from human Wharton’s jelly mesenchymal stem cells protect hippocampal neurons from oxidative stress and synapse damage induced by amyloid-β oligomers

**DOI:** 10.1186/s13287-019-1432-5

**Published:** 2019-11-20

**Authors:** Victor Bodart-Santos, Luiza R. P. de Carvalho, Mariana A. de Godoy, André F. Batista, Leonardo M. Saraiva, Luize G. Lima, Carla Andreia Abreu, Fernanda G. De Felice, Antonio Galina, Rosalia Mendez-Otero, Sergio T. Ferreira

**Affiliations:** 1 Institute of Biophysics Carlos Chagas Filho, Federal University of Rio de Janeiro, Rio de Janeiro, RJ 21941-902 Brazil; 2 Institute of Medical Biochemistry Leopoldo de Meis, Federal University of Rio de Janeiro, Rio de Janeiro, RJ 21941-902 Brazil; 3 Institute of Biomedical Sciences, Federal University of Rio de Janeiro, Rio de Janeiro, RJ 21941-902 Brazil; 4 National Cancer Institute, Rio de Janeiro, RJ 20230-240 Brazil; 5 Centre for Neuroscience Studies and Department of Psychiatry, Queen’s University, Kingston, Ontario K7L 3N6 Canada; 6 National Institute of Science and Technology for Regenerative Medicine, Rio de Janeiro, RJ 21941-590 Brazil

## Abstract

**Background:**

Mesenchymal stem cells (MSCs) have been explored as promising tools for treatment of several neurological and neurodegenerative diseases. MSCs release abundant extracellular vesicles (EVs) containing a variety of biomolecules, including mRNAs, miRNAs, and proteins. We hypothesized that EVs derived from human Wharton’s jelly would act as mediators of the communication between hMSCs and neurons and could protect hippocampal neurons from damage induced by Alzheimer’s disease-linked amyloid beta oligomers (AβOs).

**Methods:**

We isolated and characterized EVs released by human Wharton’s jelly mesenchymal stem cells (hMSC-EVs). The neuroprotective action of hMSC-EVs was investigated in primary hippocampal cultures exposed to AβOs.

**Results:**

hMSC-EVs were internalized by hippocampal cells in culture, and this was enhanced in the presence of AβOs in the medium. hMSC-EVs protected hippocampal neurons from oxidative stress and synapse damage induced by AβOs. Neuroprotection by hMSC-EVs was mediated by catalase and was abolished in the presence of the catalase inhibitor, aminotriazole.

**Conclusions:**

hMSC-EVs protected hippocampal neurons from damage induced by AβOs, and this was related to the transfer of enzymatically active catalase contained in EVs. Results suggest that hMSC-EVs should be further explored as a cell-free therapeutic approach to prevent neuronal damage in Alzheimer’s disease.

## Background

Alzheimer’s disease (AD) is responsible for 50–70% of dementia cases in the elderly, and effective treatments are still not available [1, 2]. The main clinical symptom is cognitive decline, which begins with loss of the capacity to form new memories and progresses throughout a few years to broadly impaired cognitive functions.

Converging evidence accumulated in the past 20 years indicates that soluble oligomers of the amyloid-β peptide (AβOs), toxins that accumulate in AD brains, are implicated in brain damage and dysfunction, including neuronal tau hyperphosphorylation, changes in calcium homeostasis, increased production of reactive oxygen species (ROS), mitochondrial dysfunction, and aberrant activation of microglia and astrocytes (reviewed in [3–5]). These events result in a degenerative process that leads to synapse failure and cognitive deficits in AD. Currently available therapies for AD are essentially symptomatic and not disease-modifying, thus presenting limited efficacies [6]. Therefore, there is an urgent need to develop effective ways to prevent or reverse neuronal damage associated with AD.

Mesenchymal stem cells (MSCs) from different sources have emerged as potential therapeutic alternatives for a number of neurological disorders, with promising in vivo and in vitro results in models of neurodegenerative diseases, including AD [7–11]. For example, placenta-derived MSCs have been shown to reduce brain Aβ levels, glial activation, and memory impairment in Aβ-infused mice [12]. Placenta-derived MSCs further downregulate the release of inflammatory cytokines, promoting neuronal differentiation and preventing cell death [12]. Transplantation of bone marrow-derived MSCs in Aβ-infused mice triggered microglial activation and reduced brain Aβ deposits [13], and umbilical cord blood-derived MSCs reduced Aβ deposits and tau hyperphosphorylation and improved memory in APP/PS1 transgenic mice [10, 11]. In addition, the expression of the pro-inflammatory cytokines, IL-1β and TNF-α, was decreased while the expression of the anti-inflammatory cytokine, IL-4, was increased in the brains of APP/PS1 mice after treatment with bone marrow-derived MSCs [14].

Neuroprotection by MSCs appears related to their potential to release several molecules and particles that act paracrinally to stimulate neurogenesis and/or combat inflammation [15, 16], a concept known as secretome. The MSC secretome is composed of a protein fraction, which comprises peptides, cytokines and growth factors, and a vesicular fraction, comprising extracellular vesicles (EVs) [17–20]. EVs represent a heterogeneous population of secreted vesicles, including exosomes and microvesicles originating from distinct cellular compartments [21]. EVs constitute an important mechanism of cellular communication and can facilitate the transfer of mRNAs, miRNAs, bioactive lipids, and proteins between cells without direct cell-to-cell contact [21–23]. EVs released by MSCs (MSC-EVs) have been implicated in the protective actions of these cells [24, 25], and their therapeutic potential has been demonstrated in studies employing different cell models and central nervous system (CNS) lesions (reviewed in [26, 27]). We recently reported that EVs derived from rat bone marrow MSCs prevent oxidative stress and synapse damage induced by AβOs in hippocampal neurons [9]. In the current study, we report the characterization of EVs derived from human Wharton’s jelly mesenchymal stem cells (hMSCs) and investigate their neuroprotective potential in an in vitro model of AD.

## Methods

### Ethical considerations

All procedures involving human-derived materials were approved by and followed the guidelines of the Institutional Human Ethical and Research Committee of the Clementino Fraga Filho Hospital of the Federal University of Rio de Janeiro (HUCFF/UFRJ 519.235). Experiments involving animals followed NIH guidelines and were approved by and monitored by the Institutional Animal Care and Use Committee of the Federal University of Rio de Janeiro (IBCCF 076).

### Mesenchymal stem cell cultures

Human Wharton’s jelly mesenchymal stem cells (hMSCs) were obtained from umbilical cords (20 cm in length) donated by mothers following informed consent. Cords were collected in 200 mL PBS with 1% antibiotics (100 U/ml penicillin and 100 μg/ml streptomycin; Life Technologies) and fungizone (amphotericin B, 250 μg/ml; Life Technologies) and were processed within 24 h of storage at 4 °C. After removal of the veins and artery, the jelly was crushed, suspended in 100 mL Dulbecco’s modified Eagle’s medium F-12 (DMEM F-12; Invitrogen) with 1% antibiotics, digested with type II collagenase (200 U/mL) for 16 h at 37 °C under slow agitation, and submitted to a 2000×*g* centrifugation step for 15 min. The pellet was suspended and dissociated in DMEM F-12 supplemented with 15% fetal bovine serum (FBS; Invitrogen) and 1% antibiotics, and submitted to two centrifugation steps of 15 min each at 1000×*g* and 500×*g*. Cells were plated in 75-cm^2^ flasks and maintained in a 5% CO_2_ humidified atmosphere at 37 °C. The medium was changed every 2–3 days. Cells were grown until approximately 90% confluence, trypsinized (0.25% trypsin plus 1 mM EDTA; Life Technologies), and plated again at a density of 7 × 10^3^ cells/cm^2^.

### Hippocampal cultures

Hippocampi from 18-day-old rat embryos were dissected and cultured as previously described [28, 29] with minor modifications. Briefly, hippocampi were dissected and incubated with trypsin (0.25%) in PBS at 37 °C for 5 min. Five milliliters of DMEM medium supplemented with 5% FBS, 1% antibiotics, and 1% fungizone was added and the tissue dissociated by pipetting up and down 30 times. Cells were plated on glass coverslips previously coated with poly-l-lysine (0.1 mg/ml; > 300,000 Da molecular mass; Sigma Aldrich) at a density of 377 cells/mm^2^. After 30 min, the medium was replaced by Neurobasal supplemented with 2% B27 (ThermoFisher Scientific), 1% antibiotics, 1% fungizone, and 1% Glutamax 100X (Life Technologies). Cultures were maintained at 37 °C in a humidified 5% CO_2_ atmosphere for 18–21 days in vitro. One third of the medium was replaced after 10 days.

### Isolation of hMSC-EVs

hMSCs at 90% confluence were washed twice with PBS and cultured for 24 h in serum-free DMEM F12 medium. The number of viable cells was assessed and conditioned medium (CM) was collected. CM was centrifuged at 2000×*g* for 20 min to remove cellular debris and, subsequently, at 100,000×*g* for 2 h at 4 °C (Optima L-90 K ultracentrifuge; Beckman Coulter). The pellet containing EVs was resuspended in PBS and stored at − 80 °C. For transmission electron microscopy analysis, CM was first centrifuged at 16,000×*g* for 30 min to obtain microvesicles, and then at 100,000×*g* for 2 h to obtain exosomes.

### Characterization of hMSCs and hMSC-EVs

The StemPro Differentiation Kit (Life Technologies) was used according to the manufacturer’s recommendations to determine the capacity of hMSCs to undergo adipogenic or chondrogenic differentiation. Characterization of hMSCs and hMSC-microvesicles was performed by fluorescence-activated cell sorting (FACS) utilizing a FACSCalibur™ sorter (BD Biosciences) and analyzed by CellQuest software (BD Biosciences). FITC-conjugated antibodies (anti-CD73, anti-CD90, anti-CD105, anti-CD146, anti-CD14, anti-CD34, anti-CD45, and anti-HLA-DR; BD Biosciences) were incubated with samples for 30 min in PBS containing 0.5% BSA at 4 °C. Mouse isotypic IgG was used as a control.

FACS analysis of hMSC-exosomes was performed using exosome capture beads based on anti-CD63 coupled antibody (Invitrogen Dynabeads Exosome Human CD63 Isolation/Detection Reagent) following the manufacturer’s recommendations. Specific FITC-conjugated exosomal markers (anti-CD9 and anti-CD81; BD Biosciences) were used as above.

### Nanoparticle tracking analysis

Particle density and size distribution of hMSC-EVs were measured on NanoSight NS300 (Malvern Instruments). EVs were diluted 1:1000 in PBS for analysis, and results represent averages of five 1-min readings for each sample.

### Transmission electron microscopy

hMSC-EVs were diluted 1:100 in 4% paraformaldehyde and added to 300 mesh collodium-coated copper grids (Electron Microscopy Science; Hatfield, PA) and allowed to dry for 1 h at room temperature. Grids were transferred to 1% glutaraldehyde solution for 5 min, washed with distilled water, and set to dry for 30 min. Grids were stained with 5% uranyl acetate for 5 min and washed with abundant distilled water for 3 min. Each grid was placed under a drop of 0.14% methylcellulose for 5 min and washed thoroughly in distilled water. Analyses were performed using a Jeol JEM-1011 transmission electron microscope operated at 80 kV at the Rudolf Barth Electron Microscopy Platform/Oswaldo Cruz Institute/Fiocruz (Rio de Janeiro, Brazil).

### Preparation of AβOs

AβOs were prepared as previously described [30]. Briefly, the peptide was dissolved to 1 mM in hexafluoro-2-propanol and stored in aliquots as a dried film at − 80 °C after solvent evaporation. Individual aliquots of film were resuspended in DMSO to a final concentration of 5 mM. The solution was diluted to 100 μM in ice-cold PBS and left at 4 °C overnight. The preparation was centrifuged at 14,000×*g* for 10 min at 4 °C, and the supernatant containing soluble AβOs was stored at 4 °C. Protein concentration was determined using the BCA assay (Pierce). Oligomer solutions were always used within 48 h of preparation. Characterization of each and every oligomer preparation was performed by size-exclusion HPLC chromatography and, occasionally, by Western blot using oligomer-sensitive NU4 antibody [31] (kindly provided by Prof. William L. Klein, Northwestern University). Preparations consistently comprised soluble oligomeric species including dimers, tetramers, and higher molecular mass oligomers of 50–180 kDa, ranging in diameter from 1.5 to 3.5 nm [32–35].

### Cellular uptake of hMSC-EVs and immunocytochemistry

Cultured hippocampal cells were incubated with hMSC-EVs (6 × 10^8^ particles) for 22 h after addition of vehicle (PBS containing 2% DMSO) or AβOs (500 nM) for 2 h. In uptake experiments, we employed hMSC-EVs derived from hMSCs that were double-labeled with SYTO RNASelect (for RNA labeling) and Vybrant DiI (for membrane labeling) (both from Molecular Probes). Cells were fixed with 4% paraformaldehyde/4% sucrose solution for 15 min at 4 °C. Cell nuclei were labeled with DAPI. Images were acquired on a Zeiss LSM 510 confocal microscope.

For immunocytochemistry, fixed cells were blocked with 4% bovine serum albumin at room temperature. Primary antibodies used were rabbit anti-MAP 2 (1:200; Santa Cruz), mouse anti-GLUA1 (1:200; MAB2263, Santa Cruz), and guinea pig anti-GLT-1 (1:400; AB1783, Millipore) and were incubated overnight at 4 °C. For labeling with mouse anti-synaptophysin (1:1000; Vector Labs) and rabbit anti-PSD-95 (1:200; Santa Cruz) antibodies, cells were permeabilized with 0.1% Triton X-100 (Merck) for 5 min at room temperature before blocking with 10% horse serum for 1 h. After incubation with primary antibodies, cells were washed with PBS and incubated with Alexa Fluor-conjugated secondary antibodies (1:1000; Thermo Fisher) for 2 h at room temperature. Images were acquired on a Zeiss Axio Observer Z1 microscope or a Nikon C2 confocal microscope.

### Reactive oxygen species

Formation of reactive oxygen species (ROS) was evaluated in live hippocampal neurons using 2 μM CM-H2DCFDA (Life Technologies) as previously described [9, 30]. Probe fluorescence was analyzed using NIH ImageJ software as described [30]. Briefly, hippocampal cultures were exposed to 500 nM AβOs for 2 h at 37 °C. hMSC-EVs (6.05 × 10^7^ or 1.82 × 10^8^ particles, as described in the “Results” section) were then added for 3 h and 30 min to the cultures. After this time, CM-H2DCFDA was loaded for 30 min and images were acquired on a Nikon Eclipse TE300 microscope. Nine microscopic fields were acquired using a × 20 objective per experimental condition in each of triplicate wells from three to four independent experiments using different hippocampal cultures and AβO preparations and were combined for analysis to allow quantitative estimates of changes in ROS levels. In some experiments (see the “Results” section), we used hMSC-EVs in which catalase had been inactivated by previous treatment at 37 °C with 1 mM aminotriazole for 30 min and washed twice with 20 mL PBS.

### Catalase detection

The protein content of hMSC-EVs was quantified using the Pierce BCA™ Protein Assay Kit (Thermo Fisher Scientific). One milligram of EV protein was suspended in PBS in the chamber of a high-resolution respirometer (Oroboros Oxygraph-O_2_k; Oroboros Instruments). After temperature equilibration (37 °C), the suspension received sequential H_2_O_2_ pulses corresponding to 80, 200 (2 pulses), and 400 μM peroxide, and O_2_ production resulting from the catalase-mediated breakdown of H_2_O_2_ was quantified using the DataLab 4 software coupled to the respirometer. Catalase activity (expressed as nmol O_2_ per min) was calculated from the linear phase of O_2_ formation after each pulse of H_2_O_2_. In order to confirm the role of catalase in O_2_ production, 1 mM aminotriazole (an inhibitor of the enzyme) was added.

### Statistical analyses

One-way ANOVA followed by Tukey’s post hoc test was used to compare three or more experimental conditions. *p* < 0.05 values were considered indicators of a statistically significant difference.

## Results

### Characterization of hMSCs

MSC cultures established from human umbilical cord Wharton’s jelly comprised a population of colony-forming cells with fusiform morphology (Additional file 1: Figure S1a). Cells retained the ability to commit to adipogenic or chondrogenic pathways, resulting in the accumulation of intracellular lipid droplets or active synthesis of proteoglycans, as evidenced by Oil Red O or Alcian blue staining, respectively (Additional file 1: Figure S1b, c). Consistent with an MSC-like profile, cells were positive for surface markers CD73, CD90, CD105, and CD146 and negative for hematopoietic markers HLA-DR, CD14, CD34, and CD45 (Additional file 1: Figure S1d). We further investigated the hMSC profile after culture for 24 h in serum-free medium. Serum deprivation did not affect the morphology or surface marker phenotype of hMSCs (Additional file 1: Figure S1e).

### hMSC-EVs exhibit mesenchymal surface markers and consist of a mixed population of exosomes and microvesicles

**Fig. 1:**
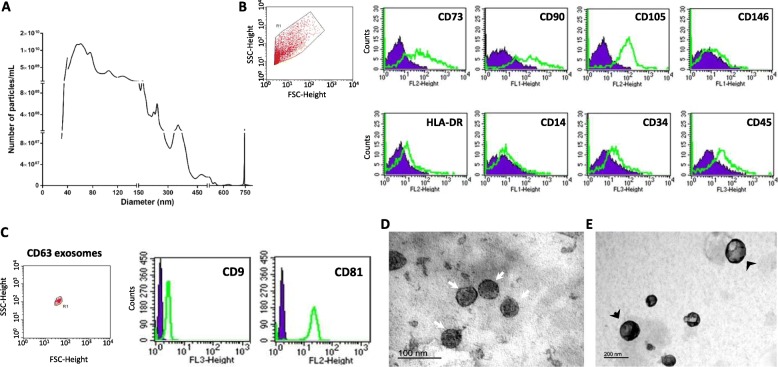
Characterization of hMSC-EVs. **a** Nanoparticle tracking analysis of hMSC-EVs indicates a mixed population of exosomes and microvesicles, comprising particles with diameters ranging between 30 and 750 nm and an average particle density of 1.7 ± 0.7 × 10^4^ particles/cell (*n* = 3 EV preparations from independent cord donors). **b** hMSC-EVs were positive for MSC markers CD73, CD90, CD105 and negative for HLA-DR, CD14, CD34, CD45, and CD146. **c** CD63-positive exosomes isolated from the total population of hMSC-EVs by CD63-conjugated capture beads are positive for exosome-associated tetraspanins, CD9 and CD81. **d**, **e** Representative TEM images of hMSC-EVs. White arrows indicate exosomes; black arrows indicate microvesicles

To characterize the ensemble of EVs secreted from hMSCs, conditioned medium from serum-deprived hMSCs was harvested. EVs were isolated and initially analyzed by nanoparticle tracking analysis (NTA). NTA revealed the presence of particles with diameters ranging from 30 to 750 nm, with a mode at 60–70 nm, consistent with a mixture of exosomes (30–100-nm-diameter particles) and microvesicles (100–750-nm-diameter particles) (Fig. 1a). Particle numbers determined by NTA for hMSC preparations derived from three independent umbilical cord donors averaged 1.7 ± 0.7 × 10^4^ particles/cell.

hMSC-EVs were positive for MSC markers CD73, CD90, and CD105, but not for CD146, HLA-DR, CD14, CD34, and CD45 (Fig. 1b). Moreover, the CD63-positive exosome fraction isolated from the total ensemble of hMSC-EVs by capture beads conjugated to the anti-CD63 antibody was positive for exosome-associated tetraspanins, CD9 and CD81 (Fig. 1c).

TEM analysis of hMSC-EVs revealed nanosized vesicles presenting roughly spherical shapes. In the fraction isolated at 100,000 g, most particles had diameters ranging from ~ 40 to 100 nm, consistent with exosomes (Fig. 1d), and an additional vesicular population with diameters greater than 150 nm was detected in the fraction isolated at 16,000 g (Fig. 1e), consistent with microvesicles.

### AβOs promote uptake of hMSCs-EVs by hippocampal cells

**Fig. 2:**
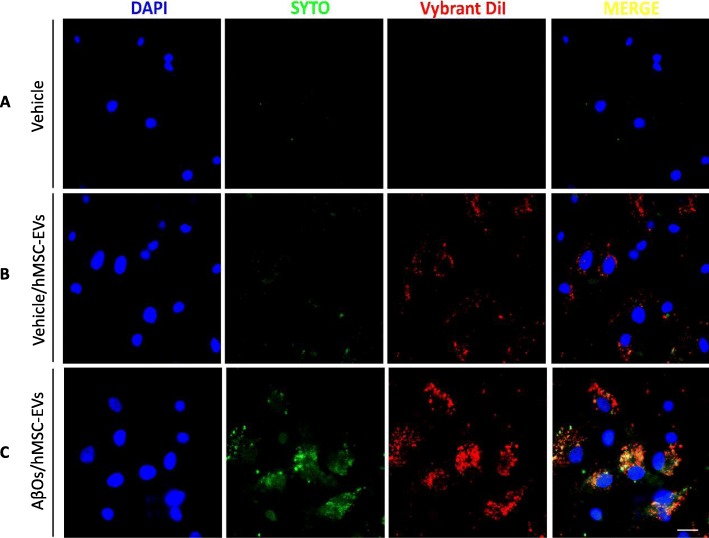
AβOs promote uptake of hMSC-EVs by hippocampal cells. Double-labeled hMSC-EVs (Vybrant DiI shown in red; SYTO RNA shown in green) were incubated for 24 h with hippocampal cultures after a previous 24-h exposure of cultures to vehicle (**b**) or 500 nM AβOs (**c**). Control hippocampal cultures in the absence of fluorescent hMSC-EVs showed no labeling (**a**). Scale bar, 20 μm

To determine whether hMSC-EVs could be internalized by cultured hippocampal cells, we used EVs isolated from hMSCs that had been double-labeled with Vybrant DiI (a membrane probe) and SYTO RNA (an RNA probe). Vehicle-exposed hippocampal cells exhibited a relatively low uptake of hMSC-EVs (Fig. 2b). Exposure of hippocampal cultures to AβOs (500 nM) for 24 h markedly increased the uptake of hMSC-EVs, as revealed by both Vybrant DiI and SYTO RNA fluorescent signals (Fig. 2c).

**Fig. 3:**
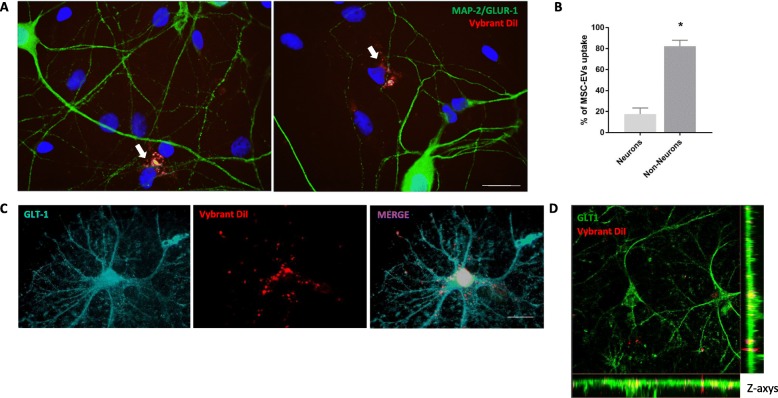
Uptake of hMSC-EVs is primarily carried out by non-neuronal cells in hippocampal cultures. **a** Vybrant DiI-labeled hMSC-EVs were incubated for 24 h with hippocampal cultures following previous incubation of cultures for 24 h with 500 nM AβOs. White arrows indicate representative uptake of hMSC-EVs by MAP 2/GluA1-positive cells. **b** Analysis of co-localization of Vibrant DiI and MAP 2/GluA1 labeling revealed that uptake of hMSC-EVs was mostly carried out by non-neuronal cells in culture. Quantification was performed on ten images from each of three coverslips in each three independent experiments. **c** GLT-1-positive astrocytes in hippocampal cultures exhibit robust hMSC-EV uptake. **d** Representative z-stack maximal intensity projection confocal image demonstrating the presence of Vybrant DiI-labeled hMSC-EVs (red) inside GLT-1-labeled astrocytes (green). Scale bar, 20 μm

Because our hippocampal cultures contain a mixture of neurons and glial cells (astrocytes and a small percentage of microglia), we sought to determine the cell type(s) responsible for uptake of hMSC-EVs. To this end, we first quantified the uptake of Vybrant diI-labeled hMSC-EVs by neurons (MAP 2/GLUR1-positive cells). We found that 18 ± 6% of hMSC-EVs labeling co-localized with MAP 2/GLUR1-positive neurons, indicating that most of the uptake was carried out by non-neuronal cells (Fig. 3a, b). We next immunolabeled cultures for glutamate transporter 1 (GLT-1), an astrocytic membrane transporter. This showed that astrocytes carried out a vigorous uptake of hMSC-EVs in hippocampal cultures (Fig. 3c). Figure 3d shows a z-stack maximal intensity projection confocal image to illustrate the presence of hMSC-EVs inside astrocytes.

### hMSC-EVs rescued AβO-induced oxidative stress in hippocampal neurons

**Fig. 4:**
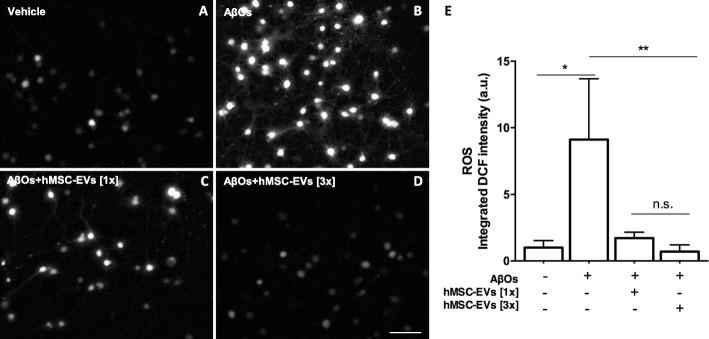
hMSC-EVs rescue AβO-induced ROS formation in hippocampal cultures. **a**, **b** Representative DCF fluorescence images from hippocampal cultures exposed to vehicle or 500 nM AβOs for a total period of 6 h. **c**, **d** Hippocampal cultures were treated with two different doses of hMSC-EVs (6.1 × 10^7^ particles, identified as [1x], or 1.82 × 10^8^ particles, identified as [3x]). Scale bar, 50 μm. **e** Integrated DCF fluorescence. Bars represent means ± SEM from three experiments using independent hippocampal culture. In each experiment, three images from each of three coverslips were acquired and analyzed using NIH ImageJ software. *p < 0.05; **p < 0.0001; one-way ANOVA followed by Tukey’s post hoc test

AβOs induce robust oxidative stress in hippocampal neurons, and this appears to be an important mechanism leading to neuronal damage [30, 36–38]. We thus asked if hMSC-EVs could reduce excessive ROS in hippocampal cultures exposed to AβOs. To this end, AβOs were added to hippocampal cultures for 2 h and hMSC-EVs were added at two different doses (6.1 × 10^7^ particles or 1.8 × 10^8^ particles; Fig. 4c, d) for an additional 4 h. Consistent with our previous reports, exposure to AβOs significantly increased ROS levels in hippocampal neurons (Fig. 4b). Treatment of cultures with both doses of hMSC-EVs reduced ROS to control levels (Fig. 4c–e).

**Fig. 5:**
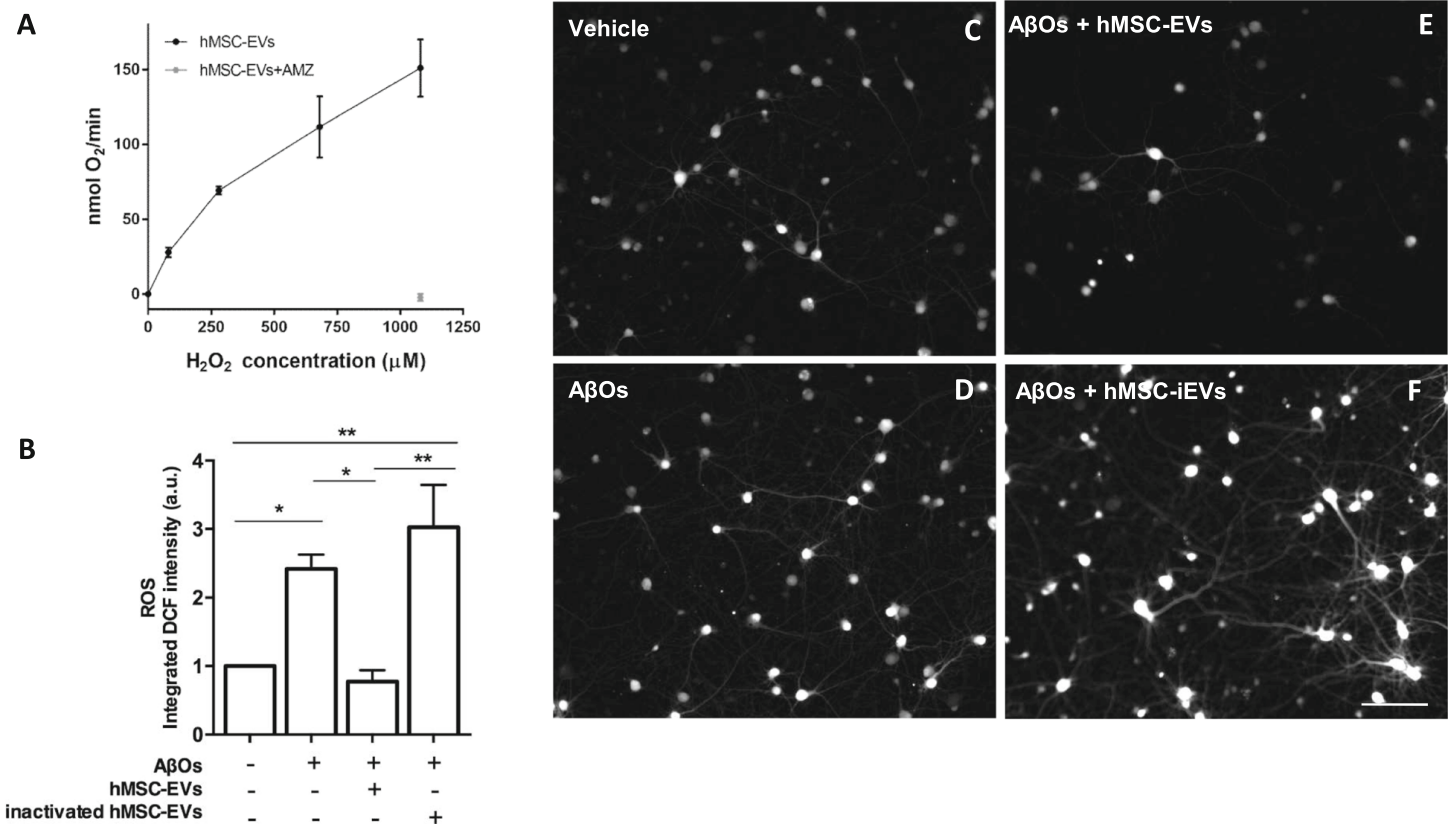
Inactivation of catalase abolishes the capacity of hMSC-EVs to block oxidative stress induced by AβOs in hippocampal cultures. **a** Catalase activity of hMSC-EVs was determined by high-resolution respirometry by measuring O_2_ release in response to increasing concentrations of added hydrogen peroxide. Treatment with aminotriazole (AMZ, a catalase inhibitor) fully inactivated catalase activity of hMSC-EVs. **b** Integrated DCF fluorescence from hippocampal cultures exposed to AbOs (or vehicle) in the presence of hMSC-EVs previously treated (or not) with aminotriazole. Bars represent means ± SEM from three experiments using independent neuronal cultures. In each experiment, three images from each of three coverslips were acquired and analyzed using NIH ImageJ software. Scale bar, 50 μm. *p < 0.05; **p < 0.0001; one-way ANOVA followed by Tukey’s post hoc test. **c**–**f** Representative DCF fluorescence images of hippocampal cultures

We recently showed that EVs derived from rat MSCs attenuate oxidative stress induced by AβOs [9] and that this anti-oxidant action was due to the presence of catalase in the EVs. In line with those observations, we found that hMSC-EVs have potent catalase activity which can be inhibited by aminotriazole, a well-known cell-permeant irreversible inhibitor of catalase activity [39] (Fig. 5a). To determine whether catalase contained in hMSC-EVs was responsible for protection against AβO-induced ROS production, we previously inhibited catalase activity of hMSC-EVs by treatment with aminotriazole before addition to hippocampal cultures. hMSC-EVs with inactivated catalase (hMSC-iEVs) were unable to prevent ROS formation instigated by AβOs in hippocampal cultures (Fig. 5a, b).

### hMSC-EVs block AβO-induced synapse damage in a catalase-dependent manner

**Fig. 6:**
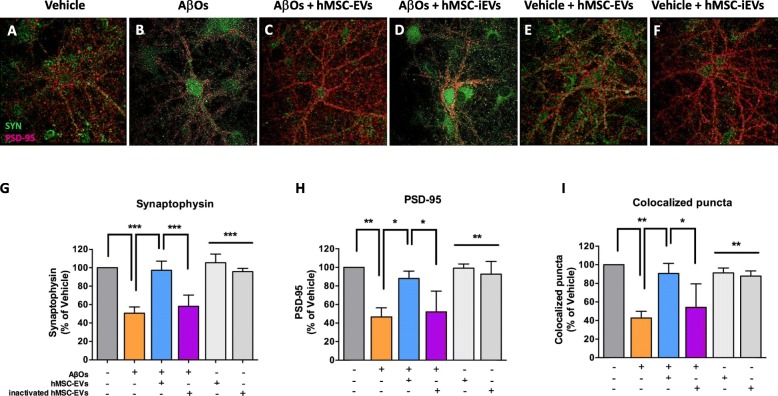
Catalase mediates the ability of hMSC-EVs to prevent synapse damage induced by AβOs. **a**, **b** Representative images from cultured hippocampal neurons exposed to 500 nM AβOs or vehicle for 24 h and immunolabeled for synaptophysin (SYP, green) and PSD-95 (red). **c**, **d** Where indicated, hippocampal cultures were treated with hMSC-EVs or hMSC-iEVs. **e**, **f** Representative images from vehicle-exposed cultures treated with hMSC-EVs or hMSC-iEVs. Integrated fluorescence intensities for synaptophysin (**g**) or PSD-95 (**h**), and numbers of co-localized synaptophysin/PSD-95 punctae (**i**). Bars represent means ± SEM from three experiments using independent cultures. Nine to 12 images from each of three coverslips were acquired and analyzed in each independent experiment. *p < 0.05; **p < 0.01; ***p < 0.001; one-way ANOVA followed by Tukey’s post hoc test. Scale bar, 20 μm

Consistent with our previous reports [38, 40, 41], hippocampal neurons exposed for 24 h to AβOs presented marked reductions in immunoreactivities for pre- and post-synaptic marker proteins, synaptophysin (Fig. 6b, g), and PSD-95 (Fig. 6b, h), respectively. A similar decrease was observed in the number of co-localized synaptophysin/PSD-95 puncta (Fig. 6b, i), indicating a reduction in synapse density in hippocampal neurons exposed to AβOs. Interestingly, treatment with hMSC-EVs for 22 h after an initial 2-h exposure to AβOs entirely prevented synapse damage (Fig. 6c, g–i). Prior inhibition of catalase activity (hMSC-iEVs) blocked the synaptoprotective action of EVs (Fig. 6d, g–i). Control experiments showed that hMSC-EVs or hMSC-iEVs did not affect synaptophysin/PSD-95 immunoreactivities or number of co-localized synaptic puncta in vehicle-exposed hippocampal neurons (Fig. 6e–i).

## Discussion

The multiple mechanisms that appear implicated in the pathogenesis of AD make it unlikely that a single therapeutic approach will be effective, suggesting that the use of combined therapies may be a viable route for treatment of AD. Several studies have indicated MSCs as promising therapeutic tools in the treatment of insults to the CNS, including spinal cord [42], cornea [43], and optic nerve injuries [44, 45]; cortical ischemia [46]; and neurodegenerative diseases, such as Huntington’s [8], Parkinson’s [47], and Alzheimer’s diseases [48]. The beneficial actions of MSCs have been attributed to their paracrine activity [49–51].

MSCs can be obtained from a variety of tissues, including the umbilical cord [52], adipose tissue [53], dental pulp [54], and bone marrow [55]. In the current study, we successfully isolated adherent cells with MSC characteristics from human Wharton’s jelly [52, 56–58]. These cells had fibroblast-like morphology, expressed mesenchymal but not hematopoietic surface markers, and effectively differentiated into adipogenic and chondrogenic lineages (Additional file 1: Figure S1).

Previous studies have described the ability of MSCs to secrete vesicles (MSC-EVs) with critical biological activities [59–61]. Cells secrete a wide range of EVs with different sizes, morphologies, and contents, which interact with target cells and modify their phenotypes and function [21, 62]. EVs play an essential role in paracrine signaling, as they represent a packaging mechanism that protects bioactive molecules against exposure and degradation in the extracellular environment, thus allowing those molecules to be transported and addressed specifically to target cells [63].

MSC-EVs are able to cross the blood-brain barrier, thus establishing an advantage over cellular therapy and an attractive therapeutic alternative for neurological and neurodegenerative diseases. For example, intravenous administration of MSC-derived exosomes in rats promoted neurite remodeling, neurogenesis and angiogenesis, leading to functional recovery in stroke [64, 65] and traumatic brain injury models [66, 67]. Furthermore, exosomes derived from dental pulp MSCs reduce 6-OHDA-induced apoptosis in dopaminergic neurons in an in vitro model of Parkinson’s disease [68], whereas EVs derived from adipose tissue MSCs express high levels of neprilysin, an important enzyme in degradation of Aβ in the brain [69]. Those EVs were efficiently internalized by neuroblastoma cells and led to decreases in intracellular and extracellular Aβ levels [69].

We showed that EVs derived from human Wharton’s jelly mesenchymal stem cells (hMSC-EVs) are composed of two vesicle populations with sizes, morphologies, and surface markers corresponding to microvesicles and exosomes. In the current study, we chose to investigate the neuroprotective actions of the entire population of EVs present in the secretome from hMSC-EVs. This choice was based on the fact that both exosomes and microvesicles have been suggested to mediate therapeutic effects of MSCs [24, 26] and on the possibility that both fractions may have complementary actions, as recently observed with other components of the MSC secretome [70]. Results showed that hMSC-EVs are internalized and transfer RNA cargoes to hippocampal cells in vitro and that this internalization is increased following exposure of hippocampal cultures to AβOs. Several studies have reported variable contents of bioactive molecules including proteins, lipids, and RNA in EVs from different sources [21], suggesting that these molecules may operate at different levels and distinct time scales in target cells.

We found that most of the uptake of hMSC-EVs in hippocampal cultures was carried out by non-neuronal cells, notably astrocytes. Astrocytes play essential roles in synaptic and brain plasticity and participate in inflammatory responses of the CNS [71]. In AD, increased astrocyte proliferation has been shown to be accompanied by changes in phenotype to a more reactive state [72, 73]. This abnormal astrocyte activation is also present in AD models involving accumulation of AβOs [74, 75]. AβOs interact with astrocytes, leading to their activation, abnormal generation of ROS, and impairment of their neuroprotective actions [76]. A recent study showed that treatment with MSC-EVs decreases activation of astrocytes and microglia, downregulates IL-1β and TNF-α pro-inflammatory cytokines, upregulates IL-4 and IL-10 anti-inflammatory cytokines, and improves memory in APP/PS1 transgenic mice [77]. Our current results highlight the capacity of astrocytes to uptake hMSCs-EVs in the presence of AβOs, and suggest that hMSC-EVs may be explored as tools to modulate astrocyte function in AD.

Brain oxidative stress is a pathological correlate of aging and AD [78, 79]. High concentrations of ROS are toxic to cells and trigger mitochondrial and DNA damage, mutations, and cytoskeletal disorganization, leading to loss of homeostasis and cell death [3, 80–85]. We have previously shown that AβOs promote neuronal oxidative stress via aberrant activation of NMDA receptors [86]. MSCs are resistant to oxidative stress and present high levels of anti-oxidant enzymes [87]. Administration of MSCs reduces oxidative stress in a rat stroke model [88] and Friedreich’s ataxia [89] models. In a recent study, catalase loaded into macrophage-derived exosomes decreased oxidative stress and increased neuronal survival in vitro and in vivo in a mouse model of Parkinson’s disease [90]. Moreover, EVs derived from human Wharton’s jelly mesenchymal stromal cells protect kidney cells from ischemia/reperfusion injury [91]. We recently showed that rat bone marrow MSCs, as well as EVs secreted from such cells, rescued hippocampal cultures from oxidative stress and synapse damage induced by AβOs [9].

In the current study, we report that hMSC-EVs decreased ROS accumulation upon exposure of hippocampal neurons to AβOs. Significantly, hMSC-EVs further prevented the decrease in synaptic proteins and synapse density induced by AβOs in hippocampal cultures. These beneficial effects were dependent on the activity of catalase contained within hMSC-EVs, as inhibition of catalase activity in hMSC-EVs abrogated their neuroprotective actions against neuronal ROS accumulation and synapse damage induced by AβOs. We have established that AβOs induce robust neuronal oxidative stress that can be detected by intracellular ROS-sensitive probes [30, 36, 92]. Therefore, our current observation of catalase-mediated protection against oxidative stress requires catalase to be transferred to and active in the cytoplasm. The notion that catalase is contained within EVs provides a straightforward mechanism to explain the transfer of the active enzyme to cells.

We have also considered the possibility that catalase could be present in soluble form in the hMSC secretome or adsorbed to the external surface of EVs. However, these possibilities appear less likely for the following reasons: First, two recent studies have failed to detect catalase in the secretome from hMSCs [93, 94]. In particular, Kehl et al. did not detect catalase in an extensive proteomic analysis of the soluble protein fraction of the secretome from human Wharton’s jelly MSCs conditioned medium [94]. This is consistent with our own results of proteomic analysis of hMSCs conditioned medium (data not shown). Second, the protocol we have used for catalase inactivation by aminotriazole includes two washes with large volumes of PBS. This should not only result in effective removal of any soluble protein but would also be expected to result in a marked reduction in proteins that might be adsorbed to the external surface of the EVs. In conclusion, our results and those in the recent literature suggest that catalase is not present in the soluble fraction of the hMSC secretome, but rather is contained within EVs, and that its transfer mediates protection against AβO-induced damage.

We further note that the protective action of hMSC-EVs we now describe was detected after a brief period of treatment with EVs. This fast time scale appears compatible with the notion that proteins (e.g., catalase) and/or other factors transferred by EVs mediate protection. One could envision that alterations in gene expression in target cells mediated by mRNA and miRNAs transferred by EVs could lead to additional, complementary protective mechanisms acting on a longer time scale.

Additionally, our findings indicate shared mechanisms of neuroprotection by EVs from either rat or human origin and also from different tissues (rat bone marrow versus human Wharton’s jelly), as observed in the present and our previous study [9]. A hypothetical mechanism of action of hMSC-EVs is that their uptake by astrocytes could reduce (via catalase activity) abnormal ROS production and propagation of astrocyte neurotoxic effects induced by AβOs. Additionally, hMSC-EVs may potentiate protective astrocyte functions on synapses, a mechanism that warrants further exploration in future studies.

## Conclusion

Our findings suggest that hMSC-EVs mediate a paracrine mechanism of neuroprotection against AβO-induced neuronal oxidative stress and synapse damage. Neuroprotection by hMSC-EVs was mediated by active catalase contained within EVs. hMSC-EVs may represent a novel therapeutic alternative to transfer biomolecules, such as RNA and proteins, which may act to reduce AD pathology and contribute to the treatment of AD and other neurodegenerative disorders.

## Supplementary information

**Additional file 1: Figure S1.** Characterization of human Wharton’s Jelly mesenchymal stem cells (hMSCs). a MSCs had fibroblast-like morphology and stemness was confirmed by adipogenic (b, Oil Red O labeling) or chondrogenic differentiation (c, Alcian blue labeling). Scale bars, 50 μm. MSC phenotype was further confirmed by flow cytometry. Cells maintained in control medium (d) or serum-free medium (e) for 24 h were immunolabeled with antibodies against HLA-DR, CD14, CD34, CD45, CD73, CD90, CD105 or CD146. Green traces correspond to fluorescence intensities of markers, while purple shaded curves indicate isotypic controls.

## Data Availability

All data generated or analyzed during this study are included in this published article and its supplementary information files.

## Abbreviations

AD: Alzheimer’s disease
AβO: Amyloid-β oligomer
CM: Conditioned medium
CNS: Central nervous system
DMEM: Dulbecco’s modified Eagle’s medium
EVs: Extracellular vesicles
FACS: Fluorescence-activated cell sorting
FBS: Fetal bovine serum
GLT-1: Glutamate transporter
hMSCs: Human Wharton’s jelly mesenchymal stem cells
hMSC-iEVs: hMSC-extracellular vesicles with catalase activity inhibited by aminotriazole
IL: Interleukin
MSCs: Mesenchymal stem cells
MSC-EVs: MSCs-extracellular vesicles
NTA: Nanoparticle tracking analysis
PBS: Phosphate-buffered saline
ROS: Reactive oxygen species
TNF-α: Tumor necrosis factor alpha

## References

[1] Anand R, Gill KD, Mahdi AA (2014). Therapeutics of Alzheimer’s disease: past, present and future. Neuropharmacology.

[2] Imtiaz B, Tolppanen AM, Kivipelto M, Soininen H (2014). Future directions in Alzheimer’s disease from risk factors to prevention. Biochem Pharmacol.

[3] Ferreira ST, Klein WL (2011). The Abeta oligomer hypothesis for synapse failure and memory loss in Alzheimer’s disease. Neurobiol Learn Mem.

[4] Mucke L, Selkoe DJ (2012). Neurotoxicity of amyloid beta-protein: synaptic and network dysfunction. Cold Spring Harb Perspect Med.

[5] Ferreira IL, Ferreiro E, Schmidt J, Cardoso JM, Pereira CM, Carvalho AL, Oliveira CR, Rego AC (2015). Abeta and NMDAR activation cause mitochondrial dysfunction involving ER calcium release. Neurobiol Aging.

[6] Lindvall O, Kokaia Z (2006). Stem cells for the treatment of neurological disorders. Nature.

[7] Gubert F, Satiago MF (2016). Prospects for bone marrow cell therapy in amyotrophic lateral sclerosis: how far are we from a clinical treatment?. Neural Regen Res.

[8] Moraes L, Vasconcelos-dos-Santos A, Santana FC, Godoy MA, Rosado-de-Castro PH, Jasmin, Azevedo-Pereira RL, Cintra WM, Gasparetto EL, Santiago MF (2012). Neuroprotective effects and magnetic resonance imaging of mesenchymal stem cells labeled with SPION in a rat model of Huntington’s disease. Stem Cell Res.

[9] de Godoy MA, Saraiva LM, de Carvalho LRP, Vasconcelos-Dos-Santos A, Beiral HJV, Ramos AB, Silva LRP, Leal RB, Monteiro VHS, Braga CV (2018). Mesenchymal stem cells and cell-derived extracellular vesicles protect hippocampal neurons from oxidative stress and synapse damage induced by amyloid-beta oligomers. J Biol Chem.

[10] Kim JY, Kim DH, Kim JH, Lee D, Jeon HB, Kwon SJ, Kim SM, Yoo YJ, Lee EH, Choi SJ (2012). Soluble intracellular adhesion molecule-1 secreted by human umbilical cord blood-derived mesenchymal stem cell reduces amyloid-beta plaques. Cell Death Differ.

[11] Lee HJ, Lee JK, Lee H, Carter JE, Chang JW, Oh W, Yang YS, Suh JG, Lee BH, Jin HK (2012). Human umbilical cord blood-derived mesenchymal stem cells improve neuropathology and cognitive impairment in an Alzheimer’s disease mouse model through modulation of neuroinflammation. Neurobiol Aging.

[12] Yun HM, Kim HS, Park KR, Shin JM, Kang AR, il Lee K, Song S, Kim YB, Han SB, Chung HM (2013). Placenta-derived mesenchymal stem cells improve memory dysfunction in an Abeta1-42-infused mouse model of Alzheimer’s disease. Cell Death Dis.

[13] Lee JK, Jin HK, Bae JS (2009). Bone marrow-derived mesenchymal stem cells reduce brain amyloid-beta deposition and accelerate the activation of microglia in an acutely induced Alzheimer’s disease mouse model. Neurosci Lett.

[14] Lee JK, Jin HK, Endo S, Schuchman EH, Carter JE, Bae JS (2010). Intracerebral transplantation of bone marrow-derived mesenchymal stem cells reduces amyloid-beta deposition and rescues memory deficits in Alzheimer’s disease mice by modulation of immune responses. Stem Cells.

[15] Noh MY, Lim SM, Oh KW, Cho KA, Park J, Kim KS, Lee SJ, Kwon MS, Kim SH (2016). Mesenchymal stem cells modulate the functional properties of microglia via TGF-beta secretion. Stem Cells Transl Med.

[16] Volkman R, Offen D (2017). Concise review: mesenchymal stem cells in neurodegenerative diseases. Stem Cells.

[17] Kim HO, Choi S-M, Kim H-S (2013). Mesenchymal stem cell-derived secretome and microvesicles as a cell-free therapeutics for neurodegenerative disorders. Tissue Eng Regenerative Med.

[18] Baez-Jurado Eliana, Hidalgo-Lanussa Oscar, Barrera-Bailón Biviana, Sahebkar Amirhossein, Ashraf Ghulam Md, Echeverria Valentina, Barreto George E. (2019). Secretome of Mesenchymal Stem Cells and Its Potential Protective Effects on Brain Pathologies. Molecular Neurobiology.

[19] Vizoso Francisco, Eiro Noemi, Cid Sandra, Schneider Jose, Perez-Fernandez Roman (2017). Mesenchymal Stem Cell Secretome: Toward Cell-Free Therapeutic Strategies in Regenerative Medicine. International Journal of Molecular Sciences.

[20] Ferreira JR, Teixeira GQ, Santos SG, Barbosa MA, Almeida-Porada G, Goncalves RM (2018). Mesenchymal stromal cell secretome: influencing therapeutic potential by cellular pre-conditioning. Front Immunol.

[21] Raposo G, Stoorvogel W (2013). Extracellular vesicles: exosomes, microvesicles, and friends. J Cell Biol.

[22] Felicetti F, De Feo A, Coscia C, Puglisi R, Pedini F, Pasquini L, Bellenghi M, Errico MC, Pagani E, Care A (2016). Exosome-mediated transfer of miR-222 is sufficient to increase tumor malignancy in melanoma. J Transl Med.

[23] Ding G, Zhou L, Qian Y, Fu M, Chen J, Chen J, Xiang J, Wu Z, Jiang G, Cao L (2015). Pancreatic cancer-derived exosomes transfer miRNAs to dendritic cells and inhibit RFXAP expression via miR-212-3p. Oncotarget.

[24] Rani S, Ryan AE, Griffin MD, Ritter T (2015). Mesenchymal stem cell-derived extracellular vesicles: toward cell-free therapeutic applications. Mol Ther.

[25] Yang Y, Ye Y, Su X, He J, Bai W, He X (2017). MSCs-derived exosomes and neuroinflammation, neurogenesis and therapy of traumatic brain injury. Front Cell Neurosci.

[26] Koniusz S, Andrzejewska A, Muraca M, Srivastava AK, Janowski M, Lukomska B (2016). Extracellular vesicles in physiology, pathology, and therapy of the immune and central nervous system, with focus on extracellular vesicles derived from mesenchymal stem cells as therapeutic tools. Front Cell Neurosci.

[27] Liew LC, Katsuda T, Gailhouste L, Nakagama H, Ochiya T (2017). Mesenchymal stem cell-derived extracellular vesicles: a glimmer of hope in treating Alzheimer’s disease. Int Immunol.

[28] De Felice FG, Vieira MN, Saraiva LM, Figueroa-Villar JD, Garcia-Abreu J, Liu R, Chang L, Klein WL, Ferreira ST (2004). Targeting the neurotoxic species in Alzheimer’s disease: inhibitors of Abeta oligomerization. FASEB J.

[29] Vieira MN, Forny-Germano L, Saraiva LM, Sebollela A, Martinez AM, Houzel JC, De Felice FG, Ferreira ST (2007). Soluble oligomers from a non-disease related protein mimic Abeta-induced tau hyperphosphorylation and neurodegeneration. J Neurochem.

[30] De Felice FG, Velasco PT, Lambert MP, Viola K, Fernandez SJ, Ferreira ST, Klein WL (2007). Abeta oligomers induce neuronal oxidative stress through an N-methyl-D-aspartate receptor-dependent mechanism that is blocked by the Alzheimer drug memantine. J Biol Chem.

[31] Lambert MP, Velasco PT, Chang L, Viola KL, Fernandez S, Lacor PN, Khuon D, Gong Y, Bigio EH, Shaw P (2007). Monoclonal antibodies that target pathological assemblies of Abeta. J Neurochem.

[32] Jurgensen S, Antonio LL, Mussi GE, Brito-Moreira J, Bomfim TR, De Felice FG, Garrido-Sanabria ER, Cavalheiro EA, Ferreira ST (2011). Activation of D1/D5 dopamine receptors protects neurons from synapse dysfunction induced by amyloid-beta oligomers. J Biol Chem.

[33] Paula-Lima AC, Adasme T, SanMartin C, Sebollela A, Hetz C, Carrasco MA, Ferreira ST, Hidalgo C (2011). Amyloid beta-peptide oligomers stimulate RyR-mediated Ca2+ release inducing mitochondrial fragmentation in hippocampal neurons and prevent RyR-mediated dendritic spine remodeling produced by BDNF. Antioxid Redox Signal.

[34] Sebollela A, Freitas-Correa L, Oliveira FF, Paula-Lima AC, Saraiva LM, Martins SM, Mota LD, Torres C, Alves-Leon S, de Souza JM (2012). Amyloid-beta oligomers induce differential gene expression in adult human brain slices. J Biol Chem.

[35] Figueiredo CP, Clarke JR, Ledo JH, Ribeiro FC, Costa CV, Melo HM, Mota-Sales AP, Saraiva LM, Klein WL, Sebollela A (2013). Memantine rescues transient cognitive impairment caused by high-molecular-weight abeta oligomers but not the persistent impairment induced by low-molecular-weight oligomers. J Neurosci.

[36] Decker H, Jurgensen S, Adrover MF, Brito-Moreira J, Bomfim TR, Klein WL, Epstein AL, De Felice FG, Jerusalinsky D, Ferreira ST (2010). N-methyl-D-aspartate receptors are required for synaptic targeting of Alzheimer’s toxic amyloid-beta peptide oligomers. J Neurochem.

[37] Saraiva Leonardo M., Seixas da Silva Gisele S., Galina Antonio, da-Silva Wagner S., Klein William L., Ferreira Sérgio T., De Felice Fernanda G. (2010). Amyloid-β Triggers the Release of Neuronal Hexokinase 1 from Mitochondria. PLoS ONE.

[38] Brito-Moreira J, Lourenco MV, Oliveira MM, Ribeiro FC, Ledo JH, Diniz LP, Vital JFS, Magdesian MH, Melo HM, Barros-Aragao F (2017). Interaction of amyloid-beta (Abeta) oligomers with neurexin 2alpha and neuroligin 1 mediates synapse damage and memory loss in mice. J Biol Chem.

[39] Nicholls P (1962). The reaction between aminotriazole and catalase. Biochim Biophys Acta.

[40] Lourenco MV, Clarke JR, Frozza RL, Bomfim TR, Forny-Germano L, Batista AF, Sathler LB, Brito-Moreira J, Amaral OB, Silva CA (2013). TNF-alpha mediates PKR-dependent memory impairment and brain IRS-1 inhibition induced by Alzheimer’s beta-amyloid oligomers in mice and monkeys. Cell Metab.

[41] Batista AF, Forny-Germano L, Clarke JR, Lyra ESNM, Brito-Moreira J, Boehnke SE, Winterborn A, Coe BC, Lablans A, Vital JF (2018). The diabetes drug liraglutide reverses cognitive impairment in mice and attenuates insulin receptor and synaptic pathology in a non-human primate model of Alzheimer’s disease. J Pathol.

[42] Zeng X, Zeng YS, Ma YH, Lu LY, Du BL, Zhang W, Li Y, Chan WY (2011). Bone marrow mesenchymal stem cells in a three-dimensional gelatin sponge scaffold attenuate inflammation, promote angiogenesis, and reduce cavity formation in experimental spinal cord injury. Cell Transplant.

[43] Roddy GW, Oh JY, Lee RH, Bartosh TJ, Ylostalo J, Coble K, Rosa RH, Prockop DJ (2011). Action at a distance: systemically administered adult stem/progenitor cells (MSCs) reduce inflammatory damage to the cornea without engraftment and primarily by secretion of TNF-alpha stimulated gene/protein 6. Stem Cells.

[44] Mesentier-Louro LA, Zaverucha-do-Valle C, da Silva-Junior AJ, Nascimento-Dos-Santos G, Gubert F, de Figueiredo AB, Torres AL, Paredes BD, Teixeira C, Tovar-Moll F (2014). Distribution of mesenchymal stem cells and effects on neuronal survival and axon regeneration after optic nerve crush and cell therapy. PLoS One.

[45] Millan-Rivero JE, Nadal-Nicolas FM, Garcia-Bernal D, Sobrado-Calvo P, Blanquer M, Moraleda JM, Vidal-Sanz M, Agudo-Barriuso M (2018). Human Wharton’s jelly mesenchymal stem cells protect axotomized rat retinal ganglion cells via secretion of anti-inflammatory and neurotrophic factors. Sci Rep.

[46] de Vasconcelos Dos Santos A, da Costa Reis J, Diaz Paredes B, Moraes L, Jasmin, Giraldi-Guimaraes A, Mendez-Otero R: Therapeutic window for treatment of cortical ischemia with bone marrow-derived cells in rats. Brain Res 2010, 1306:149–158. 10.1016/j.brainres.2009.09.094 19799881

[47] Chen D, Fu W, Zhuang W, Lv C, Li F, Wang X (2017). Therapeutic effects of intranigral transplantation of mesenchymal stem cells in rat models of Parkinson’s disease. J Neurosci Res.

[48] Harach T, Jammes F, Muller C, Duthilleul N, Cheatham V, Zufferey V, Cheatham D, Lukasheva YA, Lasser T, Bolmont T (2017). Administrations of human adult ischemia-tolerant mesenchymal stem cells and factors reduce amyloid beta pathology in a mouse model of Alzheimer’s disease. Neurobiol Aging.

[49] van Haaften T, Byrne R, Bonnet S, Rochefort GY, Akabutu J, Bouchentouf M, Rey-Parra GJ, Galipeau J, Haromy A, Eaton F (2009). Airway delivery of mesenchymal stem cells prevents arrested alveolar growth in neonatal lung injury in rats. Am J Respir Crit Care Med.

[50] Lee JW, Fang X, Krasnodembskaya A, Howard JP, Matthay MA (2011). Concise review: mesenchymal stem cells for acute lung injury: role of paracrine soluble factors. Stem Cells.

[51] Yang M, Li Q, Sheng L, Li H, Weng R, Zan T (2011). Bone marrow-derived mesenchymal stem cells transplantation accelerates tissue expansion by promoting skin regeneration during expansion. Ann Surg.

[52] Fang Z, Yin X, Wang J, Tian N, Ao Q, Gu Y, Liu Y (2016). Functional characterization of human umbilical cord-derived mesenchymal stem cells for treatment of systolic heart failure. Exp Ther Med.

[53] Lee NE, Kim SJ, Yang SJ, Joo SY, Park H, Lee KW, Yang HM, Park JB (2017). Comparative characterization of mesenchymal stromal cells from multiple abdominal adipose tissues and enrichment of angiogenic ability via CD146 molecule. Cytotherapy.

[54] Ishikawa J, Takahashi N, Matsumoto T, Yoshioka Y, Yamamoto N, Nishikawa M, Hibi H, Ishigro N, Ueda M, Furukawa K (2016). Factors secreted from dental pulp stem cells show multifaceted benefits for treating experimental rheumatoid arthritis. Bone.

[55] Hart ML, Kaupp M, Brun J, Aicher WK (2017). Comparative phenotypic transcriptional characterization of human full-term placenta-derived mesenchymal stromal cells compared to bone marrow-derived mesenchymal stromal cells after differentiation in myogenic medium. Placenta.

[56] Ranjbaran H, Abediankenari S, Mohammadi M, Jafari N, Khalilian A, Rahmani Z, Momeninezhad Amiri M, Ebrahimi P (2018). Wharton’s jelly derived-mesenchymal stem cells: isolation and characterization. Acta Med Iran.

[57] Barczewska M, Grudniak M, Maksymowicz S, Siwek T, Oldak T, Jezierska-Wozniak K, Gladysz D, Maksymowicz W (2019). Safety of intrathecal injection of Wharton’s jelly-derived mesenchymal stem cells in amyotrophic lateral sclerosis therapy. Neural Regen Res.

[58] Bharti D, Shivakumar SB, Park JK, Ullah I, Subbarao RB, Park JS, Lee SL, Park BW, Rho GJ (2018). Comparative analysis of human Wharton’s jelly mesenchymal stem cells derived from different parts of the same umbilical cord. Cell Tissue Res.

[59] Fatima F, Ekstrom K, Nazarenko I, Maugeri M, Valadi H, Hill AF, Camussi G, Nawaz M (2017). Non-coding RNAs in mesenchymal stem cell-derived extracellular vesicles: deciphering regulatory roles in stem cell potency, inflammatory resolve, and tissue regeneration. Front Genet.

[60] Borger V, Bremer M, Ferrer-Tur R, Gockeln L, Stambouli O, Becic A, Giebel B. Mesenchymal stem/stromal cell-derived extracellular vesicles and their potential as novel immunomodulatory therapeutic agents. Int J Mol Sci. 2017:18(7). 10.3390/ijms18071450 PMC5535941 28684664

[61] Crivelli B, Chlapanidas T, Perteghella S, Lucarelli E, Pascucci L, Brini AT, Ferrero I, Marazzi M, Pessina A, Torre ML (2017). Mesenchymal stem/stromal cell extracellular vesicles: from active principle to next generation drug delivery system. J Control Release.

[62] Colombo M, Raposo G, Thery C (2014). Biogenesis, secretion, and intercellular interactions of exosomes and other extracellular vesicles. Annu Rev Cell Dev Biol.

[63] Turturici G, Tinnirello R, Sconzo G, Geraci F (2014). Extracellular membrane vesicles as a mechanism of cell-to-cell communication: advantages and disadvantages. Am J Physiol Cell Physiol.

[64] Xin H, Li Y, Cui Y, Yang JJ, Zhang ZG, Chopp M (2013). Systemic administration of exosomes released from mesenchymal stromal cells promote functional recovery and neurovascular plasticity after stroke in rats. J Cereb Blood Flow Metab.

[65] Xin H, Li Y, Liu Z, Wang X, Shang X, Cui Y, Zhang ZG, Chopp M (2013). MiR-133b promotes neural plasticity and functional recovery after treatment of stroke with multipotent mesenchymal stromal cells in rats via transfer of exosome-enriched extracellular particles. Stem Cells.

[66] Kim DK, Nishida H, An SY, Shetty AK, Bartosh TJ, Prockop DJ (2016). Chromatographically isolated CD63+CD81+ extracellular vesicles from mesenchymal stromal cells rescue cognitive impairments after TBI. Proc Natl Acad Sci U S A.

[67] Zhang Y, Chopp M, Meng Y, Katakowski M, Xin H, Mahmood A, Xiong Y (2015). Effect of exosomes derived from multipluripotent mesenchymal stromal cells on functional recovery and neurovascular plasticity in rats after traumatic brain injury. J Neurosurg.

[68] Jarmalaviciute A, Tunaitis V, Pivoraite U, Venalis A, Pivoriunas A (2015). Exosomes from dental pulp stem cells rescue human dopaminergic neurons from 6-hydroxy-dopamine-induced apoptosis. Cytotherapy.

[69] Katsuda T, Tsuchiya R, Kosaka N, Yoshioka Y, Takagaki K, Oki K, Takeshita F, Sakai Y, Kuroda M, Ochiya T (2013). Human adipose tissue-derived mesenchymal stem cells secrete functional neprilysin-bound exosomes. Sci Rep.

[70] Mitchell R, Mellows B, Sheard J, Antonioli M, Kretz O, Chambers D, Zeuner MT, Tomkins JE, Denecke B, Musante L (2019). Secretome of adipose-derived mesenchymal stem cells promotes skeletal muscle regeneration through synergistic action of extracellular vesicle cargo and soluble proteins. Stem Cell Res Ther.

[71] Clarke LE, Barres BA (2013). Emerging roles of astrocytes in neural circuit development. Nat Rev Neurosci.

[72] Acosta C, Anderson HD, Anderson CM (2017). Astrocyte dysfunction in Alzheimer disease. J Neurosci Res.

[73] Liddelow SA, Guttenplan KA, Clarke LE, Bennett FC, Bohlen CJ, Schirmer L, Bennett ML, Munch AE, Chung WS, Peterson TC (2017). Neurotoxic reactive astrocytes are induced by activated microglia. Nature.

[74] Forny-Germano L, Lyra e Silva NM, Batista AF, Brito-Moreira J, Gralle M, Boehnke SE, Coe BC, Lablans A, Marques SA, Martinez AM et al: Alzheimer’s disease-like pathology induced by amyloid-beta oligomers in nonhuman primates. J Neurosci 2014, 34(41):13629–13643. 10.1523/JNEUROSCI.1353-14.2014 PMC6608380 25297091

[75] Ledo JH, Azevedo EP, Clarke JR, Ribeiro FC, Figueiredo CP, Foguel D, De Felice FG, Ferreira ST (2013). Amyloid-beta oligomers link depressive-like behavior and cognitive deficits in mice. Mol Psychiatry.

[76] Diniz LP, Tortelli V, Matias I, Morgado J, Bergamo Araujo AP, Melo HM, Seixas da Silva GS, Alves-Leon SV, de Souza JM, Ferreira ST et al: Astrocyte transforming growth factor beta 1 protects synapses against Abeta oligomers in Alzheimer’s disease model. J Neurosci 2017, 37(28):6797–6809. 10.1523/JNEUROSCI.3351-16.2017 PMC6596548 28607171

[77] Cui GH, Wu J, Mou FF, Xie WH, Wang FB, Wang QL, Fang J, Xu YW, Dong YR, Liu JR (2018). Exosomes derived from hypoxia-preconditioned mesenchymal stromal cells ameliorate cognitive decline by rescuing synaptic dysfunction and regulating inflammatory responses in APP/PS1 mice. FASEB J.

[78] Pohanka M (2014). Alzheimer’s disease and oxidative stress: a review. Curr Med Chem.

[79] Huang WJ, Zhang X, Chen WW (2016). Role of oxidative stress in Alzheimer’s disease. Biomed Rep.

[80] Walsh DM, Selkoe DJ (2004). Deciphering the molecular basis of memory failure in Alzheimer’s disease. Neuron.

[81] Morais VA, De Strooper B (2010). Mitochondria dysfunction and neurodegenerative disorders: cause or consequence. J Alzheimers Dis.

[82] Patten DA, Germain M, Kelly MA, Slack RS (2010). Reactive oxygen species: stuck in the middle of neurodegeneration. J Alzheimers Dis.

[83] Spuch C, Ortolano S, Navarro C (2012). New insights in the amyloid-beta interaction with mitochondria. J Aging Res.

[84] Leuner K, Muller WE, Reichert AS (2012). From mitochondrial dysfunction to amyloid beta formation: novel insights into the pathogenesis of Alzheimer’s disease. Mol Neurobiol.

[85] Federico A, Cardaioli E, Da Pozzo P, Formichi P, Gallus GN, Radi E (2012). Mitochondria, oxidative stress and neurodegeneration. J Neurol Sci.

[86] Brito-Moreira J, Paula-Lima AC, Bomfim TR, Oliveira FB, Sepulveda FJ, De Mello FG, Aguayo LG, Panizzutti R, Ferreira ST (2011). Abeta oligomers induce glutamate release from hippocampal neurons. Curr Alzheimer Res.

[87] Valle-Prieto A, Conget PA (2010). Human mesenchymal stem cells efficiently manage oxidative stress. Stem Cells Dev.

[88] Calio ML, Marinho DS, Ko GM, Ribeiro RR, Carbonel AF, Oyama LM, Ormanji M, Guirao TP, Calio PL, Reis LA (2014). Transplantation of bone marrow mesenchymal stem cells decreases oxidative stress, apoptosis, and hippocampal damage in brain of a spontaneous stroke model. Free Radic Biol Med.

[89] Dey R, Kemp K, Gray E, Rice C, Scolding N, Wilkins A (2012). Human mesenchymal stem cells increase anti-oxidant defences in cells derived from patients with Friedreich’s ataxia. Cerebellum.

[90] Haney MJ, Klyachko NL, Zhao Y, Gupta R, Plotnikova EG, He Z, Patel T, Piroyan A, Sokolsky M, Kabanov AV (2015). Exosomes as drug delivery vehicles for Parkinson’s disease therapy. J Control Release.

[91] Zhang G, Zou X, Miao S, Chen J, Du T, Zhong L, Ju G, Liu G, Zhu Y (2014). The anti-oxidative role of micro-vesicles derived from human Wharton-jelly mesenchymal stromal cells through NOX2/gp91(phox) suppression in alleviating renal ischemia-reperfusion injury in rats. PLoS One.

[92] De Felice FG, Vieira MN, Bomfim TR, Decker H, Velasco PT, Lambert MP, Viola KL, Zhao WQ, Ferreira ST, Klein WL (2009). Protection of synapses against Alzheimer’s-linked toxins: insulin signaling prevents the pathogenic binding of Abeta oligomers. Proc Natl Acad Sci U S A.

[93] Redondo J, Sarkar P, Kemp K, Heesom KJ, Wilkins A, Scolding NJ, Rice CM (2018). Dysregulation of mesenchymal stromal cell antioxidant responses in progressive multiple sclerosis. Stem Cells Transl Med.

[94] Kehl D, Generali M, Mallone A, Heller M, Uldry AC, Cheng P, Gantenbein B, Hoerstrup SP, Weber B (2019). Proteomic analysis of human mesenchymal stromal cell secretomes: a systematic comparison of the angiogenic potential. NPJ Regen Med.

