# Supplementary material for: Extracellular vesicles derived from human Wharton’s jelly mesenchymal stem cells protect hippocampal neurons from oxidative stress and synapse damage induced by amyloid-β oligomers
Source: Stem Cell Res Ther. 2019 Nov 20;10:332. doi: 10.1186/s13287-019-1432-5 (PMC6864996; doi:10.1186/s13287-019-1432-5)
Supplement: Supplementary file 1 — Additional file 1: Figure S1. Characterization of human Wharton’s Jelly mesenchymal stem cells (hMSCs). a MSCs had fibroblast-like morphology and stemness was confirmed by adipogenic (b, Oil Red O labeling) or chondrogenic differentiation (c, Alcian blue labeling). Scale bars, 50 μm. MSC phenotype was further confirmed by flow cytometry. Cells maintained in control medium (d) or serum-free medium (e) for 24 h were immunolabeled with antibodies against HLA-DR, CD14, CD34, CD45, CD73, CD90, CD105 or CD146. Green traces correspond to fluorescence intensities of markers, while purple shaded curves indicate isotypic controls. [file 13287_2019_1432_MOESM1_ESM.pptx]

## Slide 1
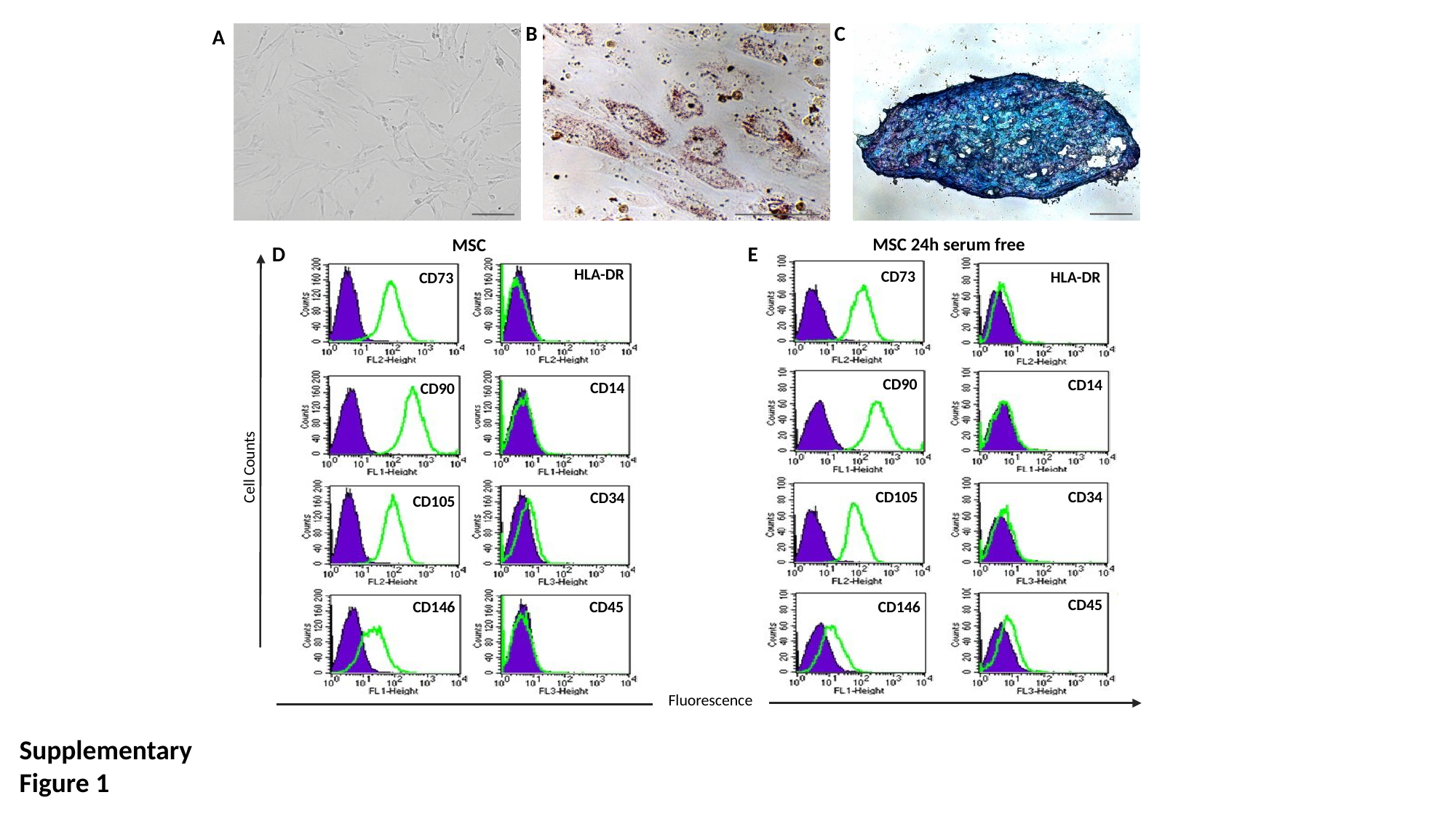

B
C
A
MSC 24h serum free
MSC
D
E
HLA-DR
CD73
HLA-DR
CD73
CD90
CD14
CD14
CD90
Cell Counts
CD34
CD105
CD34
CD105
CD45
CD146
CD45
CD146
Fluorescence
Supplementary Figure 1
